# Development of a Multi‐task Graph Neural Network for Alzheimer’s Disease Drug Repurposing

**DOI:** 10.1002/alz.089679

**Published:** 2025-01-09

**Authors:** Kang‐Lin Hsieh, German Plascencia‐Villa, Ko‐Hong Lin, George Perry, Xiaoqian Jiang, Yejin Kim

**Affiliations:** ^1^ University of Texas Health Science Center at Houston, Houston, TX USA; ^2^ The University of Texas at San Antonio (UTSA), San Antonio, TX USA; ^3^ The University of Texas at San Antonio, San Antonio, TX USA; ^4^ The University of Texas Health Science Center at Houston, Houston, TX USA

## Abstract

**Background:**

Developing drugs for treating Alzheimer’s disease (AD) has been extremely challenging and costly due to limited knowledge on underlying biological mechanisms and therapeutic targets. Repurposing drugs or their combination has shown potential in accelerating drug development due to the reduced drug toxicity while targeting multiple pathologies.

**Method:**

To address the challenge in AD drug development, we developed a multi‐task machine learning pipeline to integrate a comprehensive knowledge graph on biological/pharmacological interactions and multi‐level evidence on drug efficacy, to identify repurposable drugs and their combination candidates

**Result:**

Using the drug embedding from the heterogeneous graph representation model, we ranked drug candidates based on evidence from post‐treatment transcriptomic patterns, mechanistic efficacy in preclinical models, population‐based treatment effect, and Phase 2/3 clinical trials. We experimentally validated the top‐ranked candidates in neuronal cells, identifying drug combinations with efficacy in reducing oxidative stress and safety in maintaining neuronal viability and morphology. Our neuronal response experiments confirmed several biologically efficacious drug combinations.

**Conclusion:**

This methodology showed that harmonizing heterogeneous and complementary data/knowledge, including human interactome, transcriptome patterns, experimental efficacy, and real‐world patient data shed light on the drug development of complex diseases.

